# Operative Corridors in Endoscopic Skull Base Tumor Surgery

**DOI:** 10.3390/brainsci14030207

**Published:** 2024-02-23

**Authors:** A. Karim Ahmed, Nicholas R. Rowan, Debraj Mukherjee

**Affiliations:** 1 Department of Neurosurgery, Johns Hopkins Medical Institutions, Johns Hopkins School of Medicine, Baltimore, MD 21287, USA; aahmed33@jhmi.edu; 2 Department of Otolaryngology, Head and Neck Surgery, Johns Hopkins School of Medicine, Baltimore, MD 21287, USA

**Keywords:** endoscopic skull base surgery, transsphenoidal, transplanum, transtuberculum, transorbital, sellar, suprasellar, clivus, transpterygoid

## Abstract

Advances in technology, instrumentation, and reconstruction have paved the way for extended endoscopic approaches to skull base tumors. In the sagittal plane, the endonasal approach may safely access pathologies from the frontal sinus to the craniocervical junction in the sagittal plane, the petrous apex in the coronal plane, and extend posteriorly to the clivus and posterior cranial fossa. This review article describes these modular extended endoscopic approaches, along with crucial anatomic considerations, illustrative cases, and practical operative pearls.

## 1. Introduction

An understanding of nasal and paranasal sinus anatomy is of paramount importance for both neurosurgeons and otolaryngologists in the endonasal approach to skull base pathologies. Endoscopic endonasal approaches were traditionally confined to paranasal sinus operations and pituitary adenomas [1]; however, with significant advances in technology, instrumentation, reconstruction, and the development of novel corridors over the past decade, the endonasal approach to the skull base is now able to safely access pathologies from the frontal sinus to the craniocervical junction in the sagittal plane, the petrous apex in the coronal plane, and posteriorly to the clivus and posterior cranial fossa [1,2,3,4,5]. Congenital lesions include nasal glial heterotopia, seromucinous hamartomas, encephalocele, inflammatory pseudotumor, sinonasal papilloma, polyps, and pituitary adenomas. Inflammatory lesions in this region include allergic fungal sinusitis rhinoscleroma, granulomatosis with polyangiitis, and eosinophilic granuloma and myospherulosis. Malignancy of this lesion most commonly affects the maxillary sinus, followed by the nasal cavity, nasopharynx, and ethmoid sinuses. These include epithelial-derived neoplasms such as squamous cell carcinoma, with poorly differentiated carcinoma likely arising from inverted papillomas; sinonasal undifferentiated carcinoma (SNUC), for which induction treatment is the mainstay of initial treatment; SMARCB1-deficient sinonasal carcinoma; nasopharyngeal carcinoma; HPV-related carcinoma; and salivary/non-salivary type adenocarcinomas. Mesenchymal benign tumors include glomangiopericytoma, pyogenic granuloma, myxoma, and nasopharyngeal angiofibroma—with rich blood supply commonly from the maxillary artery. Malignant mesenchymal tumors are far less frequent, including rhabdomyosarcoma, fibrosarcoma, synovial sarcoma, hemangioendothelioma, angiosarcoma, chordoma (arising from the embryological notochord), and chondrosarcoma (arising from the petroclival synchondroses). Neuroectodermal tumors comprise olfactory neuroblastoma (from the olfactory neuroepithelium), olfactory carcinoma, and Ewing family tumors. Neuroendocrine tumors, melanoma, lymphoproliferative disorders, metastatic disease, and ameloblastoma (of enamel origin) may also affect the skull base with decreasing frequency [4,5].

Endoscopic endonasal approaches may be ideally suited for ventral skull base lesions, with the ability to visualize critical anterior neurovascular structures without brain retraction and with reduced overall surgical morbidity [2]. This review article outlines the anatomic considerations and technical nuances of the endoscopic endonasal approach to the sellar, suprasellar, medial orbital apex, anterior cranial base, and transpterygoid corridors.

## 2. Materials and Methods

A thorough review of the literature was utilized to include all clinical and anatomic, endoscopic, and endonasal skull base approaches performed in the anterior, middle, or posterior fossa using CINAHL, PubMed, Web of Science from 1980 to 2023 in addition to the review of references in eligible articles. Studies included those describing the extended transfrontal, transplanum, transtuberculum, transcribiform, transsphenoidal, transclival, transpterygoid, and transorbital approaches for skull base tumors. Critical anatomic considerations and technical limitations are described based on a review of the literature and the authors’ own experience. Illustrative cases were included for each approach as appropriate.

## 3. Results and Discussion

### 3.1. Sellar/Suprasellar

#### 3.1.1. Anatomic Considerations

In the endonasal corridor, the Agger Nasi cells are encountered relatively early during operative dissection. Latin for ‘nasal mound’, these are the anterior-most ethmoidal air cells, located anterior to the frontal recess of the frontal sinus [6,7]. The turbinates are laterally situated osseous shelves which function to regulate airflow and humidification, and if removed excessively can lead to Empty nose syndrome—characterized by a paradoxical feeling of nasal obstruction [8]. The inferior turbinate originates from the maxillary and palatine bone, delineating the inferior meatus which drains the nasolacrimal duct. The middle turbinate, of the ethmoid bone, demarcates the middle meatus and attaches superiorly to the cribiform plate and laterally to the lamina paprycea at the basal lamella, separating anterior and posterior ethmoidal air cells. The superior turbinate, also of the ethmoid bone, delineates the superior meatus just anteroinferior to the sphenoethmnoidal recess [6,7,8,9]. The various lamellae attach laterally to the lamina paprycea, forming the medial orbital wall. From anterior to posterior, these include lamella of the uncinate process, lamella of the ethmoid bulla (the largest air cell of the ethmoid sinus), lamella of the middle turbinate (basal lamella), lamella of the superior turbinate, and finally the sphenoid sinus [10]. The osteomeatal complex allows for airflow and mucociliary drainage of the paranasal sinuses, providing drainage of the frontal sinus, anterior ethmoidal air cells, maxillary sinus, and middle meatus. It is composed of the maxillary ostium, infundibulum, ethmoid bulla, uncinate process, and hiatus semilunaris [9,10,11]. As a critical component of the osteomeatal complex, the uncinate process forms the anterior–inferior border of the hiatus semilunaris, which drains the frontal recess, maxillary sinus, and anterior ethmoidal air cells. Located between the superior turbinate and sphenoid sinus, the sphenoethmoidal recess drains the posterior ethmoidal air cells and sphenoid sinus [10,11,12,13]. The sphenoid ostium may be found medial to the superior turbinate and approximately 1.5 cm superior to the choana, which demarcates the nasopharynx [14,15].

Kiesselbach’s plexus supplies blood to the anterior nasal septum, receiving contributions from the superior labial anterior ethmoidal, greater palatine, and sphenopalatine arteries. The sphenopalatine artery is the main contributor to the blood supply of the nose. As a terminal branch of the internal maxillary artery, the artery originates along the lateral rostrum of the sphenoid sinus, exiting the sphenopalatine foramen. Notably, the posterior septal artery, branching from the sphenopalatine artery, is located horizontally in the sphenoethmoidal recess between the sphenoid ostium and choana to supply the septal mucosa and floor [16].

A detailed understanding of the osseous anatomy based on preoperative imaging is critical. As an important point, laterally projecting intersinus septation of the sphenoid sinus is directed toward one of the internal carotid arteries in 85% of cases [17]. Based on its ossification pattern, the sphenoid sinus may be described as conchal, presellar, sellar, or postsellar. One must also be mindful of the presence of sphenoethmoidal air cells, as Onodi Cells, located superolateral to the sphenoid sinus, may be in close proximity to the optic nerve [14,15]. The lateral opticocarotid recesses (LOCR) become important landmarks for identification of the optic nerve and anterior cavernous genu of the internal carotid artery, and these landmarks corresponds to the optic strut of the anterior clinoid. As such, a pneumatized anterior clinoid may be appreciated in the endonasal view by a deeper, more distinct LOCR [11,14]. The medial opticocarotid recess (MOCR), usually located approximately 5.6 mm medial to LOCR and just superior to the middle clinoid process, is the medial junction of the paraclinoid internal carotid artery (ICA) and optic nerve [15]. Careful preoperative planning should be undertaken to identify the presence of a carotid ring at the middle clinoid process prior to attempted removal. Anterior and superior to the tuberculum sellae, the sphenoid limbus may be visualized as an anteriorly projecting groove, serving as the anterior boundary of the chiasmatic sulcus and a landmark to identify the location of the optic chiasm [11,14,15,18].

The diaphragma sellae, a two-layer continuation of dural folds from the roof of the cavernous sinus, forms an incomplete roof over the pituitary gland with a central defect allowing for passage of the infundibulum [19]. The pituitary gland has a rich blood supply from the superior hypophyseal, inferior hypophyseal, and McConnell capsular arteries of the internal carotid arteries. Further contributors include the infundibular (from posterior communicating artery) and prechiasmatic (from the ophthalmic artery) arteries. As an important distinction, pituitary transposition frequently involves identification and careful sacrifice of the inferior hypophyseal arteries, which may be safely done; by contrast, the superior hyphophyseal arteries should be preserved whenever possible, as they supply the anterior pituitary gland, optic chiasm, and proximal optic nerves. The medial wall of the cavernous sinus is comprised of a single dural layer, separate from the pituitary capsule, with several parasellar ligaments that anchor the medial wall [20]. These include the caroticoclinoid ligament, superior parasellar ligament, inferior parasellar ligament, and posterior parasellar ligament [21].

Arising from the diaphragma sellae anteriorly, the Liliequist membrane consists of two sheets separating the suprasellar/chiasmatic cistern from the interpeduncular cistern attached to the mamillary bodies, and the interpeduncular cistern from the prepontine cistern at the junction of the midbrain and pons. The chiasmatic cistern, situated between the sella and hypothalamus, contains the proximal Sylvian veins, optic chiasm, anterior communicating artery complex, and infundibulum [18,19,20].

#### 3.1.2. Illustrative Case

**Figure 1:**
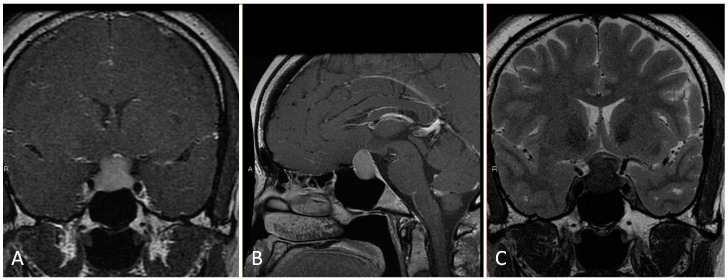
Preoperative imaging of a 38-year-old patient with bitemporal hemianopsia and a sellar lesion consistent with Rathke’s cleft cyst. (**A**) Coronal T1 post-contrast MRI. (**B**) Sagittal T1 post-contrast MRI. (**C**) Coronal T2 MRI.

The present case is a 38-year-old female who presented with irregular menses and menorrhea. Lab work demonstrated a mild elevation in serum prolactin (54) consistent with mild stalk effect, along with normal adrenal, growth hormone, and thyroid axis labs. Imaging demonstrated a complex sellar lesion (Figure 1). The patient was started on cabergoline with improvement of her amenorrhea. Her visual fields examination was consistent with bitemporal, left worse than right, hemianopsia, and she agreed to proceeding with surgical resection for diagnosis and decompression of her optic apparatus.

The patient was placed supine with Mayfield cranial fixation. Both nares were decongested with two epinephrine-soaked pledgets bilaterally. Once there was adequate vasoconstriction, the inferior and middle turbinates were outfractured bilaterally. An incision was made at the caudal edge of the septum. A Cottle elevator was used to identify the submucoperichondrial plane from which mucosa was elevated from the posterior septum. A caudal strut was demarcated at the posterior aspect of the dissection with an incision and crossover cut developed with a Cottle elevator. The deviated portion of the septum was resected and removed. On the right, the superior turbinate was visualized, and its inferior one-third was resected. The sphenoid ostium was identified and cannulated. With visualization into the sphenoid sinus, a superior nasoseptal rescue flap incision was made with monopolar cautery and the mucosa was reflected inferiorly off the nasal septum, sphenoid rostrum, and arch of the choanae to protect the posterior septal artery pedicle. A posterior septectomy was then completed. Using the natural os bilaterally, the sphenoidotomies were enlarged and the entire face of the sphenoid was removed, visualizing the entire sphenoid sinus, sella, tuberculum, planum, and LOCRs.

**Figure 2:**
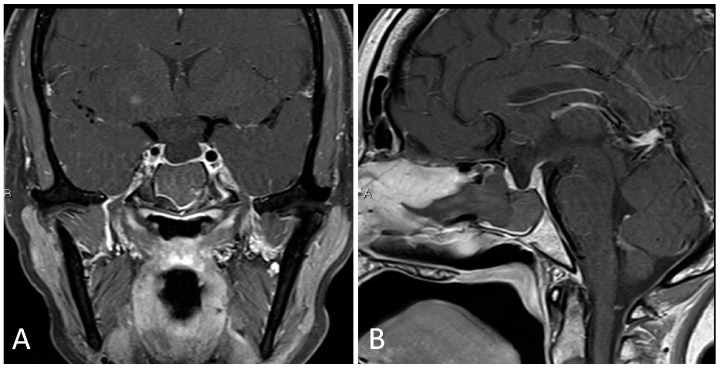
Postoperative imaging consistent with gross total resection of Rathke’s cleft cyst. (**A**) Coronal T1 post-contrast MRI. (**B**) Sagittal T1 post-contrast MRI.

The sella was then thinned using a combination of high-speed drilling with a diamond drill bit, with the thinned shell of sellar bone subsequently peeled away from the dura using Kerrison rongeurs. The dura was opened low in the sella in an inverted horizontal and linear fashion. Fibrinous, caseous yellow debris was immediately encountered and sent for frozen and permanent pathological specimen—all consistent with Rathke’s cleft cyst. Resection was performed with bimanual suctions sweeping laterally on the floor of the sella and moving upwards until the entire cyst was decompressed. The diaphragma was noted to descend following decompression, and no cerebrospinal fluid (CSF) leak was encountered. Following hemostasis, gelfoam was placed in the nasal corridor to help ensure the mucosa would remain moist during the peri-operative period, but the opening to the cyst was purposefully left unobstructed to help minimize risk of cyst recurrence. The patient was discharged home two days later without endocrine dysfunction, with a postoperative MRI demonstrating excellent decompression (Figure 2), and with recovery to full visual fields by her 2-month postoperative follow-up ophthalmology visit.

#### 3.1.3. Practical Pearls

- An understanding of the osseous anatomy of the nasal corridor, paranasal sinuses, septum, intersinus septations, and relevant vascular structures are of paramount importance in the approach to sellar lesions.
- A pedicled nasoseptal rescue flap may be useful to obtain during the approach to the sellar lesions, particularly in cases of possible CSF leak, and should take into account the horizontal orientation of the posterior septal branch of the sphenopalatine artery.
- Dural opening of Rathke’s cleft cysts, one should begin anteriorly and inferiorly to minimize the risk of inadvertent CSF leak and iatrogenic injury to the pituitary gland.
- One must be mindful of diaphragm downward migration and identification during cyst resection to avoid a CSF leak.

#### 3.1.4. Illustrative Case

**Figure 3:**
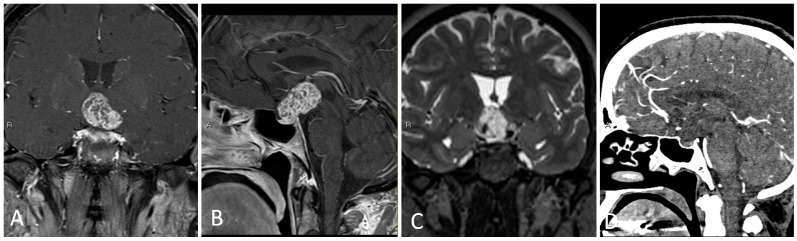
Preoperative imaging of a 56-year-old patient with an enhancing, partially calcified sellar and suprasellar lesion consistent with craniopharyngioma. (**A**) Coronal T1 post-contrast MRI. (**B**) Sagittal T1 post-contrast MRI. (**C**) Coronal T2-weighted MRI. (**D**) Sagittal thin cut CTA head demonstrating partial peripheral calcification.

This is the case of a 57-year-old female who presented with 2 months of worsening vision loss in bitemporal fields, left worse than right, with imaging demonstrating a large cystic sellar and suprasellar lesion extending to the third ventricle (Figure 3). Admission labs were notable for mild central hypothyroidism. Given the large size of the suprasellar lesion with mass effect on the optic apparatus and vision symptom, the decision was made to proceed with surgical resection.

Given the proximity of the lesion to the third ventricle, a high-flow CSF leak was anticipated, and a lumbar drain was therefore placed at the beginning of the case. Following lumbar drain placement, the patient was placed supine with Mayfield cranial fixation. The abdomen and lateral thigh were prepped for possible fat and fascia lata grafts, respectively. Both nares were decongested with two epinephrine-soaked pledgets bilaterally. Once there was adequate vasoconstriction, the inferior and middle turbinates were outfractured bilaterally. The superior turbinate was visualized on the right and its inferior third was resected. The sphenoid os was identified and a broad, right sided, pedicled nasoseptal flap was created with an anterior incision, parallel to the inferior border of the middle turbinate, transitioning superiorly along the dorsum and posteriorly to the caudal strut. Subsequently, an inferior incision was made along the vomer to the nasal floor, at the junction of the hard and soft palate, to the inferior meatus. This incision was extended to join the superior incision at the caudal strut, and the flap was raised in a circumferential fashion, with care to preserve the vascularized pedicle. Using the posteroinferior border of the uncinate process, the maxillary ostium was identified and a maxillary antrostomy was performed, alongside a total ethmoidectomy. A septoplasty was completed, a rescue flap incision was made on the left side, and a modest posterior septectomy was performed. An intersinus septation was identified coursing to the left ICA, and this septation was carefully drilled down. The bone over the sella and tuberculum sellae was carefully removed, the superior intercavernous sinus was coagulated and cut, and the dura was opened in a linear horizontal fashion at the level of the sphenoid limbus. Following sharp dissection through a soft, grey capsule located in the suprasellar cistern, gelatinous, yellow, proteinaceous material was immediately encountered and was debulked using teardrop suctures. Frozen pathological assessment of this tissue was compatible with calcified craniopharyngioma. The resection was continued superiorly into the third ventricle with removal of the posterior capsule of the tumor. The cerebral aqueduct into the fourth ventricle and bilateral foramen of Monroe were visualized. Once fenestrated into the ventricle, the remaining capsular portions of residual tumor were carefully resected eccentric to the right and left of the midline along the bilateral thalami. The eccentric portion of tumor to the left was noted to extend superiorly and inferiorly around the optic apparatus, incorporating a portion of the infundibulum, and these aspects of tumor were carefully dissected and resected, taking particular care not to manipulate the optic apparatus itself. By the end of resection, the optic nerves, superior hypophyseal arteries, and infundibulum were visualized without injury and free of tumor.

**Figure 4:**
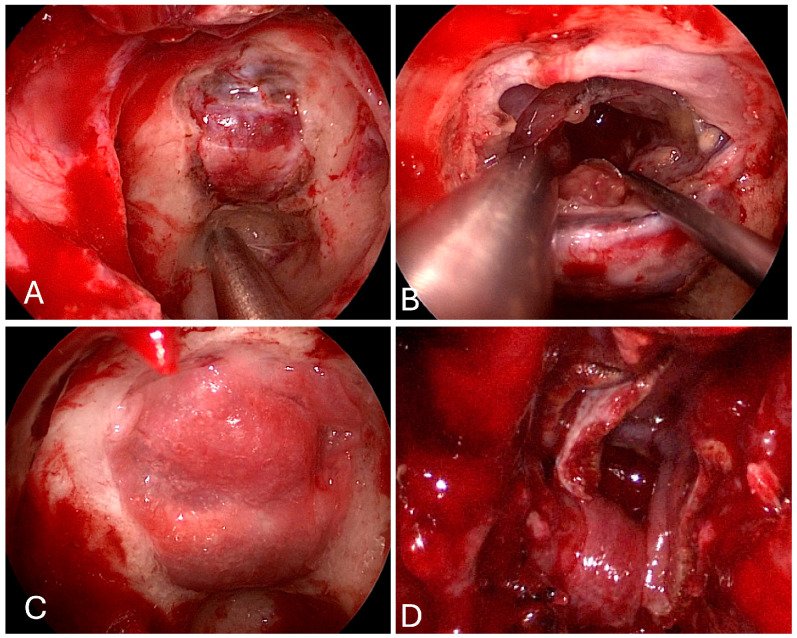
Intraoperative imaging of craniopharyngioma resection. (**A**) Sellar exposure. (**B**) Tumor resection. (**C**) Sellar onlay reconstruction. (**D**) Nasoseptal flap reconstruction.

**Figure 5:**
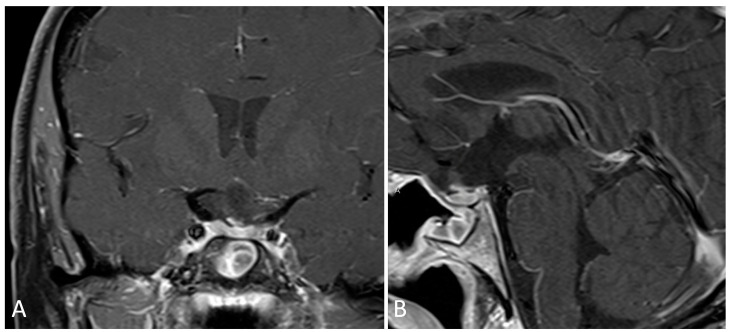
Postoperative imaging consistent with gross total resection of craniopharyngioma. (**A**) Coronal T1 post-contrast MRI. (**B**) Sagittal T1 post-contrast MRI.

Following hemostasis, a multilayer closure, including a dural substitute inlay, dural substitute onlay, and an onlay nasoseptal flap, was created at the operative site (Figure 4). The lumbar drain was kept open to drain postoperatively at a rate of 10 cc per hour; the drain was clamped on postoperative day three, and it was removed on postoperative day. She was slowly mobilized after her drain was clamped, and she was discharged home without complication on postoperative day six. Final pathology was confirmed to be adamantinomatous craniopharyngioma, and postoperative imaging demonstrated gross total resection (Figure 5). At her 6-month postoperative ophthalmology follow-up appointment, she was noted to have significant improvement in visual fields compared to her preoperative state, with no pituitary insufficiency on endocrine follow up.

#### 3.1.5. Practical Pearls

- Placement of a lumbar drain is advised at the start of surgery, or preoperatively, in cases where a high-flow CSF leak is anticipated, in order to reduce immediate postoperative intracerebral pressure (ICP) and offload excessive pressure on the skull base graft.
- Upfront harvest of a nasal septal flap may be useful for skull base reconstruction in cases of anticipated high flow CSF leak.
- The sphenoid limbus serves as an important landmark for the location of the optic nerves, bridging medial to the optic canal and forming the anterior border of the prechiasmatic sulcus.
- When approaching lesions within the suprasellar region, one should be mindful of the location of the optic apparatus (i.e., pre-fixed/post-fixed chiasm) as well as relevant neurovascular structures in the chiasmatic, lamina terminalis, interpeduncular, and prepontine cisterns.
- Craniopharyngiomas may be fenestrated into a natural CSF space, such as the basal cisterns and third ventricle, to reduce long-term cystic reaccumulation/recurrence.

### 3.2. Orbital Apex

#### 3.2.1. Anatomic Considerations

The optic nerve is approximately 5 cm in length and comprised of four segments: intraocular (1 mm), intraorbital (2.5–3 cm), intracanalicular, and prechiasmatic [22,23]. As an extension of the central nervous system, the optic nerve is surround by all three meningeal layers (dura, arachnoid, and pia mater) with an outer periosteal layer, the periorbita, which is continuous with the intracranial periosteal dura [22]. The average length of the bony orbit is 4 cm from base to orbital apex, separated by approximately 2.5 cm between the medial orbital wall from one side to the other. Lesions of the orbit are best characterized as intraconal/extraconal, relative to the muscular cone formed by the extraocular muscles, and intradural/extradural, relative to the periorbita, with orbital fat located in both the intraconal and extraconal spaces [24].

The bony orbit forms a pyramid comprising seven bones: frontal, ethmoid, lacrimal, sphenoid, zygomatic, palatine, and maxillary. The lamina paprycea of the ethmoid bone forms most of the medial orbital wall, articulating inferiorly with the orbital process of the palatine bone and maxilla, superiorly with the orbital plate of the frontal bone, posteriorly with the body of the sphenoid, and anteriorly with the lacrimal bone. The medial orbital rim is formed largely by the frontal process of the maxilla. The orbital roof is formed by a combination of the orbital plates of the frontal bone and the lesser wing of the sphenoid, which contain bony defects in both plates to form the anterior and posterior ethmoidal canals when joined. These canals demarcate the bulbar, retrobulbar, and apical segments of the orbit. The lateral orbital wall is formed by the greater wings of the sphenoid and the orbital surface of the zygomatic bone. The orbital floor is formed by the orbital process of the palatine bone and the orbital process of the maxillary bone. The lacrimal fossa is bordered anteriorly by the lacrimal crest of the maxillary bone and posteriorly by the lacrimal crest of the lacrimal bone, joining to form the lacrimomaxillary suture. Situated at the anteromedial and inferior portion of the orbit, the lacrimomaxillary suture serves as the site of insertion of the common canaliculus into the lacrimal sac via the valve of Rosenmuller to prevent reflux from the sac [24,25,26,27,28].

Blood supply to the orbit is primary through the ophthalmic artery, which passes superomedially over the intraorbital optic nerve, with several branches including the lacrimal, supraorbital, anterior ethmoidal, posterior ethmoidal, internal palpebral, supratrochlear, dorsal nasal, central retinal, anterior ciliary, posterior ciliary (long and short), central retinal, supraorbital, medial palpebral, and muscular arteries. The primary venous drainage pathway of the orbit is through the superior ophthalmic vein, draining further into the cavernous sinus, with minor tributaries to the angular and facial veins [29].

At the orbital apex, thickening of the periorbita helps to form the annulus of Zinn—a tendinous attachment for the rectus muscles, levator palpebrae superioris, and superior oblique muscles. The annulus contains the optic canal (with optic nerve and ophthalmic artery), the nasociliary nerve (V1), abducens nerve, and both superior and inferior divisions of the oculomotor nerve. The superior orbital fissure, separated from the optic canal by the optic strut, is an important landmark separating the intracranial cavernous sinus from the orbit [26,27,28].

The endoscopic endonasal approach allows for access to the medial orbit in the entire retrobulbar space through the annulus of Zinn and apex, limited medially the ophthalmic artery and optic nerve. In this approach, two working triangular corridors, superior and inferior, are separated by the medial rectus muscle to the superior oblique and inferior rectus muscles, respectively. The ophthalmic artery and optic nerve may be readily visualized and are the lateral limit of this approach. In the superior corridor, the retro-bulbar medial branches of the ophthalmic artery (i.e., anterior ethmoidal, posterior ethmoidal, central retinal artery) may be readily visualized; these structures are not easily accessible from the inferior corridor [30,31]. Superior and inferior oculomotor innervation is often on the ventral and medial surface of the extraocular muscles, and they are relatively well protected during gentle retraction in this approach. The superior division of the oculomotor nerve innervates the superior rectus and medial rectus muscles, with the inferior oblique and inferior rectus muscles innervated by the inferior division of the nerve. The trochlear nerve innervates the superior oblique muscle, passing extraconal from lateral to medial outside the annulus of Zinn, and then coursing superiorly above the superior rectus and levator palpebrae superioris muscles prior to reaching its point of innervation [25,26,27,28,29]. Medial decompression of the orbital apex in proximity to the annulus, therefore, should proceed with caution, in order to identify and preserve the trochlear nerve.

#### 3.2.2. Illustrative Case

**Figure 6:**
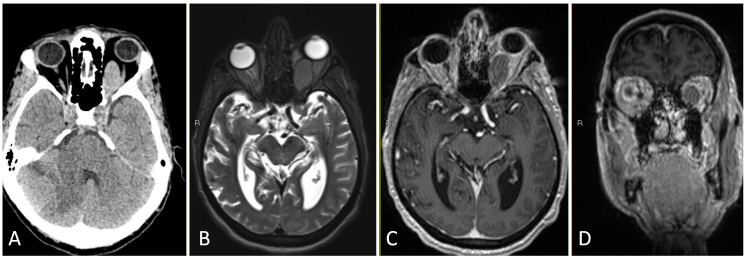
Preoperative imaging of a 62-year-old male with known metastatic neuroendocrine tumor presenting with left eye proptosis and a left intraconal medial orbital apex lesion with optic nerve compression. (**A**) Axial CT without contrast. (**B**) Axial T2-weighted MRI. (**C**) Axial T1 post-contrast MRI. (**D**) Coronal T1-post-contrast MRI.

This is the case of a 62-year-old male with a past medical history of diffusely metastatic pancreatic neuroendocrine tumor, who presented with new onset left eye proptosis over 1 week and acute blindness in the left eye over 1 day. Imaging demonstrated a left intraconal lesion concerning for metastatic disease with optic nerve compression (Figure 6).

**Figure 7:**
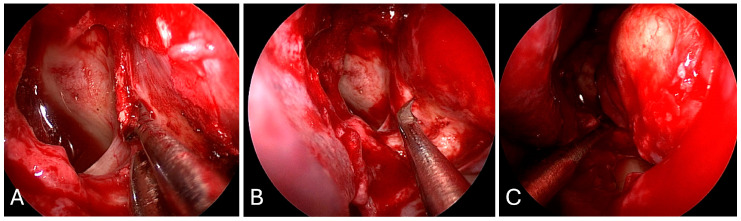
Intraoperative imaging demonstrating endoscopic endonasal transorbital approach. (**A**) Lamina paprycea is thinned and removed, exposing medial periorbita. (**B**) Periorbita is sharply incised with a sickle knife. (**C**) Tumor is exposed and debulked with suction and bipolar cautery.

Given the acuity of his vision loss and optic nerve compression, the patient was taken urgently to the operating room for endoscopic endonasal decompression of the medial orbit and pathologic diagnosis. His mean arterial pressure (MAP) was kept above 80 mm Hg and he was started on methylprednisolone for compressive optic neuropathy. The nose was decongested with epinephrine-soaked pledgets unilaterally, and the inferior and middle turbinate were fractured bilaterally. The left middle turbinate was gently medialized and the middle meatus was packed with epinephrine-soaked pledgets to ensure vasoconstriction. The uncinate process was identified and reflected anteriorly, with removal of the uncinate process revealing the maxillary ostium. A wide maxillary antrostomy was performed, followed by removal of the ethmoid bullae. The basal lamella separating anterior and posterior ethmoidal air cells was opened flush against the skull base and sphenoid, which was broadly opened to expose the orbital apex (Figure 7).

**Figure 8:**
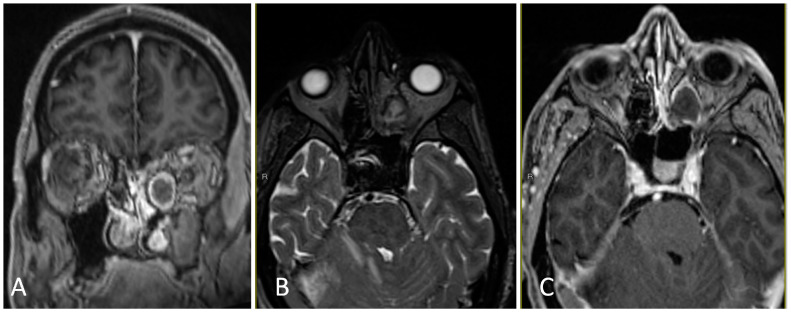
Postoperative imaging demonstrating complete resect of a left intraconal metastatic lesion and endoscopic endonasal optic nerve decompression. (**A**) Coronal T1 post-contrast MRI. (**B**) Axial T2-weighted MRI. (**C**) Axial T1 post-contrast MRI.

The mucosa over the lamina paprycea was dissected and the lamina was fractured away from the orbit from anterior to posterior. The orbital strut was downfractured to reveal part of the orbital floor. The periorbita was opened parallel to the medial rectus, and the tumor was encountered between the medial rectus and inferior rectus muscles. The necrotic tumor was debulked and suctioned with decompression of the optic apparatus. Following tumor resection, there was noted mild herniation of orbital fat with an immediate decrease in left eye proptosis. No packing was used. Postoperative imaging demonstrated medial decompression of the optic nerve and orbital apex with no compression of the optic apparatus (Figure 8). He was discharged home two days after surgery with subjective improvement in left eye vision.

#### 3.2.3. Practical Pearls

- The goals of surgical resection for metastatic lesions to the orbit are to obtain decompression of the optic apparatus and to obtain a diagnosis.
- The periorbita should be opened parallel to the medial rectus muscle to minimize inadvertent injury.
- In the medial endonasal approach, the two corridors include between the superior oblique and medial rectus muscles, as well as between the medial rectus and the inferior rectus muscles.

### 3.3. Anterior Cranial Base

#### 3.3.1. Anatomic Considerations

The ethmoid bone comprises the anterior two-thirds of the anterior cranial fossa, with the planum sphenoidale forming the posterior third. The crista galli separates the olfactory nerves in the cribiform plate and serves as an important point of attachment for the falx cerebri. Underneath the cribiform plate, the ethmoidal labyrinth is separated from the anterior cranial fossa by fovea ethmoidalis from the orbital process of the frontal bone, which attaches to the vertical lateral lamella [32,33,34]. The depth of the olfactory fossa formed by the lateral lamella may be described by the Keros classification, where Type 1 is a depth of 1–3 mm, Type 2 is 4–7 mm, and Type 3 is 8–16 mm; an increased incidence of postoperative adverse events occur with higher Keros grades [35].

The anterior ethmoidal foramen, located posterior to the frontal recess, contains the anterior ethmoidal artery, anterior ethmoidal nerve, and anterior ethmoidal vein. Similarly, the posterior ethmoidal foramen contains the posterior ethmoidal artery, posterior ethmoidal nerve, and posterior ethmoidal vein. The anterior and posterior ethmoidal nerves are extraconal branches of the nasociliary nerve of V1 (ophthalmic division of the trigeminal nerve). The anterior ethmoidal artery courses between the superior oblique and medial rectus muscles, giving a nasal branch and meningeal branch to the anterior falcine artery as well as supplying the medial and inferior dura of the anterior fossa. The posterior ethmoidal artery supplies the dura of the planum, anterior clinoid process, and chiasmatic sulcus. The anterior ethmoidal artery is approximately 24 mm from the anterior lacrimal crest and 12 mm from the posterior ethmoidal artery, which is approximately 6 mm from the optic canal [32,33]. Ligation and sectioning of the anterior and posterior ethmoidal arteries should be done close to midline to avoid inadvertent artery retraction into the orbit and subsequent development of a retro-orbital hematoma [31,32,33,34].

The attachment of the uncinate process superiorly has direct implications for frontal sinus drainage. As such, when the uncinate process attaches medial to the middle turbinate, it functions as the medial wall of the frontal recess and directs drainage of the sinus to the middle meatus via the ethmoid infundibulum. When the uncinate process is attached more laterally at the lamina paprycea, it functions as the lateral wall of the frontal recess and drains directly into the middle meatus. In such cases, the ethmoid infundibulum forms a blind recess, termed the recessus terminalis [36,37].

Following completion of a Draf III frontal sinusotomy inclusive of removal of the cribiform plate and dural opening, one is able to identify the gyrus rectus located medial and adjacent to the olfactory sulcus, with the orbital frontal gyri located laterally. The blood supply to this region is via the A2 anterior cerebral artery (ACA) branches, including primarily the fronto-polar and fronto-orbital branches [32,33,34].

#### 3.3.2. Illustrative Case

**Figure 9:**
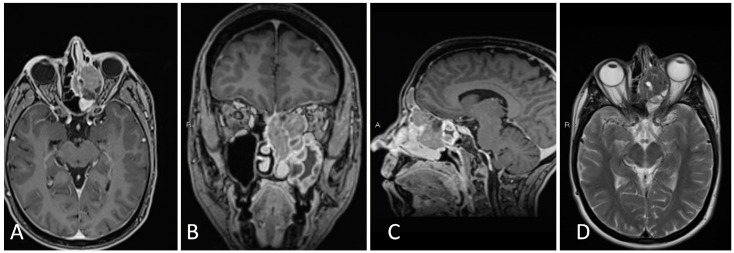
Preoperative imaging of a 44-year-old female with biopsy-proven squamous cell carcinoma of the left ethmoid, including invasion into the skull base and left lamina paprycea. (**A**) Axial T1 post-contrast MRI. (**B**) Coronal T1 post-contrast MRI. (**C**) Sagittal T1 post-contrast MRI. (**D**) Axial T2-weighted MRI.

This is the case of a 44-year-old female with a past medical history of non-Hodgkins lymphoma, who presented with 4 months of left sided facial pain and left sided headaches with nasal congestion. On imaging, she was found to have an enhancing lesion in the left ethmoid sinus with extension to the nasal cavity, lamina paprycea, and anterior fossa floor (Figure 9). Endoscopic biopsy was consistent with squamous cell carcinoma, and PET/CT demonstrated hyperactive lymph nodes in the left neck and right inguinal region. Core biopsy of the hyperactive lymph nodes were negative for malignancy.

**Figure 10:**
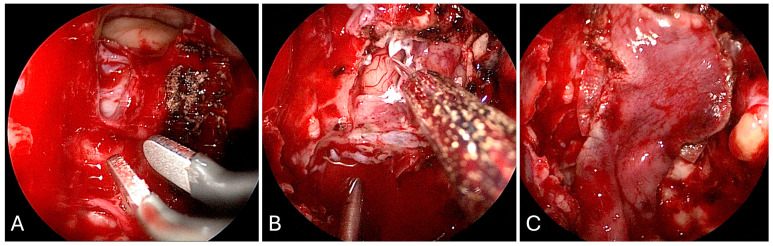
Intraoperative endoscopic endonasal anterior fossa approach. (**A**) Frontal sinusotomy with coagulation of the anterior and posterior ethmoidal arteries. (**B**) Draf III completed with opening of the dura over gyrus rectus and tumor removal. (**C**) Nasoseptal flap reconstruction.

**Figure 11:**
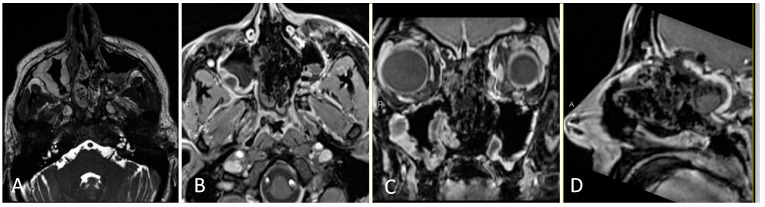
Postoperative imaging demonstrating gross total resection of a sinonasal squamous cell carcinoma. (**A**) Axial T2 high-resolution constructive interference steady state (CISS) T2-weighted MRI. (**B**) Axial T1 post-contrast MRI. (**C**) Coronal T1 post-contrast MRI. (**D**) Sagittal T1 post-contrast MRI.

Given an anticipated CSF leak, a lumbar drain was placed at the beginning of the case. The nose was decongested with epinephrine-soaked pledgets bilaterally. The entire left sinonasal airway was noted to be filled with tumor on initial endoscopy, therefore the right middle turbinate was resected upfront for increased access. A maxillary antrostomy, total ethmoidectomy, and sphenoidotomy were performed on the right side. Given likely tumor involvement of the nasal septum, a nasal septal flap was designed on the right side, incorporating the pedicle of the posterior septal artery and floor of the nasal cavity. Transitioning to the left side, the tumor was debulked circumferentially with meticulous inspection of the borders for any obvious signs of invasion. The tumor was dissected from the orbit without issue and with normal appearing mucosa underlying the lesion. A Draf III frontal sinusotomy was performed, including removal of the frontal sinus floor between the orbits, anterosuperior nasal septum, frontal beak, and frontal intersinus septum. A total ethmoidectomy was performed with coagulation and sectioning of the anterior and posterior ethmoidal arteries close to midline. The cribiform plate was removed with cuts laterally on the right and left ethmoid roof, anteriorly at the frontal sinusotomy, and posteriorly at the planum sphenoidale. The dura of the anterior fossa was sharply opened at the periphery of the anterior fossa defect, and the olfactory bulbs were resected and sent for pathology alongside the dural specimen. Following circumferential removal of the tumor, a large dural substitute inlay and onlay were placed on the anterior fossa defect. The nasal septal flap was rotated from the nasopharynx and bolstered with packing material. Doyle splints were placed on the residual nasal septum (Figure 10). Postoperatively, her lumbar drain was opened to drain 5–10 cc every hour for 48 h prior to being clamped and eventually removed. Postoperative MRI demonstrated gross total resection (Figure 11). She was discharged home in good clinical condition and completed adjuvant chemoradiation consisting of 67 cGy of intensity-modulated radiation therapy (IMRT) to the resection bed atop weekly cisplatin.

#### 3.3.3. Practical Pearls

- Anterior and posterior ethmoidal arteries should be ligated and cut close to midline in order to avoid retraction of these vessels into the orbit.
- The inlay dural substitute should be oversized as part of the multi-layered closure.
- Reconstruction with a nasoseptal flap should take into account sites of involved disease, which cannot be used in the flap.

### 3.4. Transpterygoid

#### 3.4.1. Anatomic Considerations

The transpterygoid approach allows for more lateral access than the traditional midline endoscopic endonasal approach, which is limited laterally by the anterior genu of the ICA. This expanded approach allows for access to pathologies in the lateral recess of the sphenoid sinus, petrous apex, infratemporal fossa, middle fossa floor, and posterior fossa (Table 1). In the endoscopic endonasal approach, a maxillary antrostomy and removal of the medial posterior wall of the maxillary sinus exposes the pterygopalatine fossa [38,39,40]. The pterygopalatine fossa is a crucial skull base structure, particularly in cases of malignancy, with communications to the nasal cavity through the sphenopalatine foramen, infratemporal fossa through the pterygomaxillary fissure, orbit through the inferior orbital fissure, Meckel’s cave through the foramen rotundum, lacerum segment of the ICA through the vidian canal, nasopharynx through the palatovaginal canal, and palate through the greater and lesser palatine canals. Formed by the palatine bone, pterygoid plates, and maxilla, this fossa additionally contains pterygopalatine fat surrounding the ganglion, the infraorbital branch of the maxillary artery, the infraorbital nerve from the maxillary nerve (V2) traveling towards the infraorbital foramen, and emissary veins with several tributaries [41,42,43].

The sphenopalatine artery traverses medially into the nasal cavity via the sphenopalatine foramen, formed by the orbital and sphenoid processes of the palatine bone; this artery must be cauterized and cut via the transpterygoid approach, precluding a vascularized nasoseptal flap on the ipsilateral side. The pterygopalatine ganglion is posteriorly tethered by the vidian nerve, which may be sectioned if needed (resulting in decreased lacrimation) to lateralize the ganglion and its contents. Lateral displacement of the ganglion may also put the palatine nerves on stretch, resulting in numbness of the palate. In cases where the vidian nerve is intended to be preserved, drilling of the medial pterygoid plate should remain below the border of the vidian canal and bony removal from the pterygoid wedge should be performed between foramen rotundum (superolaterally) and the vidian canal (inferomedially), which may be readily identified by following the maxillary and vidian nerves posteriorly, respectively [42,43,44].

Access to the masticator space requires detaching the pterygoid muscles from the pterygoid plates, with access to the post-styloid parapharyngeal space of the infratemporal fossa requiring complete resection of the plates. The lateral pterygoid muscle may be laterally detached to identify foramen ovale and V3, which descends adjacent the lateral pterygoid plate. The approach to Meckel’s cave requires drilling of the pterygoid wedge and lateral pterygoid plate, following the infraorbital nerve posteriorly to the maxillary nerve to skeletonize foramen rotundum [45,46]. The ‘quadrangular space’, bordered by the maxillary nerve laterally and the vertical petrous ICA medially, may be opened to expose Meckel’s cave [47]. The approach to the petrous apex requires identification of the clivus below the sphenoid floor, with dissection of pharyngobasilar fascia and overlying soft tissue medial to the Eustachan tube. Careful skeletonization of the vidian nerve leads to the lacerum segment of the ICA, a critical landmark in this region, and subsequent access to pathologies of the petrous apex. The petrous corridor can be laterally limited by the Eustachian tube, and the associated torus tubarus may be resected in cases of malignancy to increase lateral exposure. An alternative approach described to reach laterally along the petrous apex toward the internal acoustic canal (IAC) is the contralateral transmaxillary approach (CTM), which does not require Eustachian tube manipulation or dissection of the ICA [48].

**Table 1:** Landmark studies in the modular approach to endonasal skull base tumor surgery.

Authors	Approach	Study Inclusion	Outcomes
Fong et al. [49]	Sellar	Meta-analysis including eleven studies comprised of 3941 patients undergoing expanded endoscopic endonasal resection for pituitary tumors were evaluated.	Progression-free survival was significantly higher in patients who underwent gross-total resection compared to subtotal resection. Postoperative radiotherapy was associated with improved progression-free survival in patients with residual disease.
Lee et al. [50]	Suprasellar	25 articles comprising 554 pediatric patients undergoing endoscopic endonasal resection for suprasellar pathologies were included. Most common pathologies included craniopharyngiomas, adenomas, and Rathke’s cleft cysts.	No significant difference in primary tumor etiology with rates of CSF leak postoperatively (8.6% overall), including craniopharyngiomas (10.6%), adenomas (6.5%), and Rathke’s cleft cysts (7.2%).
Dubal et al. [51]	Orbital Apex	39 studies comprising 71 patients who underwent endoscopic endonasal resection of orbital apex pathologies were included.	Exclusively intraconal and extraconal pathologies included 51% and 30.6% of the cohort, respectively. Tumor etiology was most commonly cavernous hemangioma (45.1%), with 76.2% of complications as transient and no difference in rates of complication between intraconal or extraconal pathologies. There was only a 4.2% rate of recurrence in the pooled cohort.
Nicolai et al. [52]	Anterior Fossa	Exclusive EEA for anterior fossa sinonasal malignancies was performed in 134 patients with the remaining 50 undergoing a cranioendosopic combined approach. Malignancies included adenocarcinoma, squamous cell carcinoma, olfactory neuroblastoma, mucosal melanoma, and adenoid cystic carcinoma.	5-year disease-specific survival was 91.4% and 58.8% for EEA and CEA groups, respectively.
Hanna et al. [53]		120 patients were included with esthesioneuroblastoma, sarcoma, adenocarcinoma, melanoma, and squamous cell carcinoma. 93 select patients underwent exclusively expanded EEA and 27 underwent cranioendoscopic approach with combined open craniotomy.	No significant differences between combined nor exclusive EEA groups, with 5- and 10-year survival rates of 87% and 80%, respectively.
Battaglia et al. [46]	Transpterygoid	37 consecutive patients with who underwent transmaxillary transpterygoid approach for skull base tumor resection of the nasopharynx, middle fossa, or infratemporal fossa were included. Primary pathologies included juvenile nasopharyngeal angiofibroma, trigeminal schwannoma, cavernous hemangioma, adenoid cystic carcinoma, mucoepidermoid carcinoma, squamous cell carcinoma, adenocarcinoma, chondrosarcoma, and undifferentiated nasopharyngeal carcinoma.	One patient suffered internal carotid artery injury, and eight patients received adjuvant treatment. At most recent follow-up (30 months for malignant tumors and 60 months for benign tumors) all patients had stable disease without recurrence. Two patients of the cohort had stable intracranial disease, including one with a meningioma and one with adenoid cystic carcinoma.

#### 3.4.2. Illustrative Case

**Figure 12:**
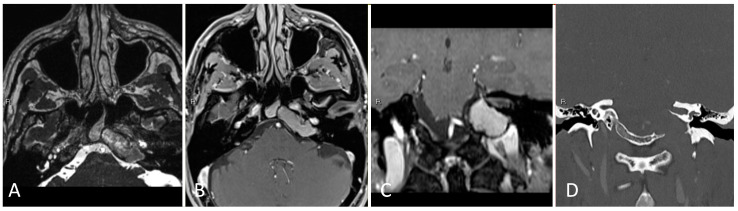
Preoperative imaging of a 43-year-old male with a left petroclival cholesterol granuloma. (**A**) Axial T2 high-resolution constructive interference steady state (CISS) T2-weighted MRI. (**B**) Axial T1 post-contrast MRI. (**C**) Coronal T1 post-contrast MRI. (**D**) Coronal CTA with contrast.

This is the case of a 43-year-old male with a past medical history of left petrous apex cholesterol granuloma that was discovered after diagnostic workup for self-resolved diplopia, atop longstanding tinnitus and chronic sinusitis. Imaging demonstrated a 3.7 × 1.5 × 2.5 cm lesion of the left petrous apex with expansile high T1 and heterogeneous mixed T2 signal intensity in the left clival and petrous apex lesion, consistent with cholesterol granuloma (Figure 12).

The nose was decongested with epinephrine-soaked pledgets bilaterally, the inferior and middle turbinates were out-fractured bilaterally, and the left middle turbinate was resected to improve visualization. A wide sphenoidotomy was then performed, a left-sided rescue mucosal flap was created, and a right-sided vascularized nasoseptal flap was created. A modestly sized posterior septectomy was performed, and the rostrum was isolated and partially resected. A generous maxillary antrostomy was created on the left side in anticipation of possible extension of the lesion lateral to the left paraclival carotid artery.

**Figure 13:**
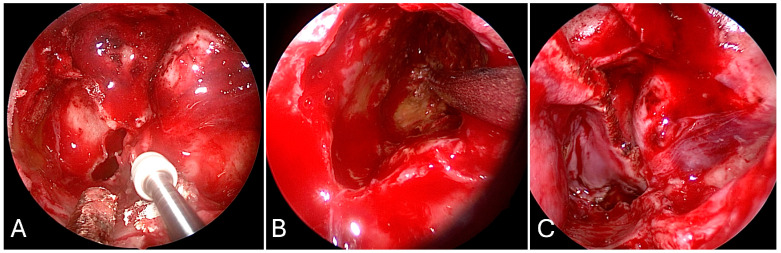
Intraoperative imaging of modified Denker’s maxillary antrostomy and transpterygoid approach to petrous apex cholesterol granuloma. (**A**) Back wall of the maxillary sinus is opened to access the pterygopalatine fossa. (**B**) Transpterygoid approach with suction resection of the granuloma. (**C**) Nasoseptal flap reconstruction.

**Figure 14:**
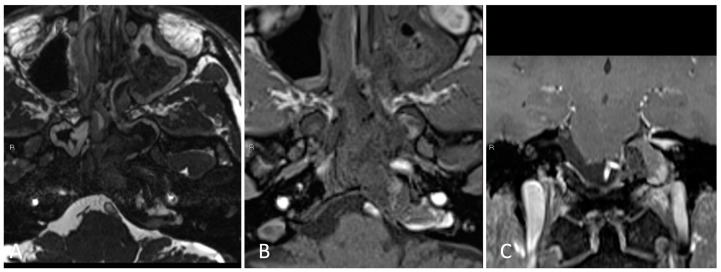
Postoperative imaging demonstrating decompression of a petrous apex cholesterol granuloma. (**A**) Axial T2 high-resolution constructive interference steady state (CISS) T2-weighted MRI. (**B**) Axial T1 post-contrast MRI. (**C**) Coronal T1 post-contrast MRI.

The mucosa was stripped from the clival recess and the intersinus septation was drilled between the well-pneumatized clival carotid protuberances. The clivus was opened medial to the left clival carotid artery to immediately encounter the cholesterol granuloma, which was sharply opened. The contents of the lesion were fully removed. The mucoperiosteum overlying the petrous internal carotid artery was similarly decompressed and visualized. The nasoseptal flap was laid into the resection cavity in an effort to allow for marsupialization of the cavity, and gelfoam was placed around this site in an attempt to minimize postoperative nasal crusting (Figure 13). Postoperative imaging demonstrated decompression of the granuloma (Figure 14) and the patient was discharged home in good condition on postoperative day two.

#### 3.4.3. Practical Pearls

- Sacrifice of the sphenopalatine artery in the transpterygoid approach precludes an ipsilateral nasoseptal flap.
- Skeletonization of the maxillary nerve leads to the ‘quadrangular space’ and Meckel’s cave.
- Skeletonization of the vidian nerve leads to the lacerum segment of the ICA.
- The contralateral transmaxillary approach may be a useful back up option for lateral petrous pathologies approaching the IAC.

## Data Availability

No data were created as part of this project.
